# Deletion of the *Lmna* gene in fibroblasts causes senescence-associated dilated cardiomyopathy by activating the double-stranded DNA damage response and induction of senescence-associated secretory phenotype

**DOI:** 10.20517/jca.2022.14

**Published:** 2022-06-10

**Authors:** Leila Rouhi, Gaelle Auguste, Qiong Zhou, Raffaella Lombardi, Melis Olcum, Kimia Pourebrahim, Sirisha M. Cheedipudi, Saman Asghar, Kui Hong, Matthew J. Robertson, Cristian Coarfa, Priyatansh Gurha, Ali J. Marian

**Affiliations:** 1 Center for Cardiovascular Genetics, Institute of Molecular Medicine, The University of Texas Health Science Center, Houston, TX 77030, USA.; 2 Department of Cardiovascular Medicine and Jiangxi Key Laboratory of Molecular Medicine, The Second Affiliated Hospital of Nanchang University, Nanchang 330006, Jiangxi, China.; 3 Division of Cardiology, Department of Advanced Biomedical Sciences, Federico II University, Naples 80100, Italy.; 4 10x Genomics, San Francisco, CA 94111, USA.; 5 Department of Molecular and Cellular Biology, Baylor College of Medicine, Houston, TX 77030, USA.

**Keywords:** Fibroblasts, senescence, fibrosis, apoptosis, lamin A/C, heart failure, cardiomyopathy

## Abstract

**Introduction:**

Mutations in the *LMNA* gene, encoding Lamin A/C (LMNA), are established causes of dilated cardiomyopathy (DCM). The phenotype is typically characterized by progressive cardiac conduction defects, arrhythmias, heart failure, and premature death. DCM is primarily considered a disease of cardiac myocytes. However, LMNA is also expressed in other cardiac cell types, including fibroblasts.

**Aim:**

The purpose of the study was to determine the contribution of the fibroblasts to DCM caused by LMNA deficiency.

**Methods and Results:**

The *Lmna* gene was deleted by crossing the platelet-derived growth factor receptor α-Cre recombinase (*Pdgfra-Cre*) and floxed *Lmna* (*Lmna*^F/F^) mice. The LMNA protein was nearly absent in ~80% of the cardiac fibroblasts and ~25% of cardiac myocytes in the *Pdgfra-Cre:Lmna*^F/F^ mice. The *Pdgfra-Cre:Lmna*^F/F^ mice showed an early phenotype characterized by cardiac conduction defects, arrhythmias, cardiac dysfunction, myocardial fibrosis, apoptosis, and premature death within the first six weeks of life. The *Pdgfra-Cre:Lmna*^wild type/F^ (*Lmna*^W/F^) mice also showed a similar but slowly evolving phenotype that was expressed within one year of age. RNA sequencing of LMNA-deficient and wild-type cardiac fibroblasts identified differential expression of ~410 genes, which predicted activation of the TP53 and TNFA/NFκB and suppression of the cell cycle pathways. In agreement with these findings, levels of phospho-H2AFX, ATM, phospho-TP53, and CDKN1A, markers of the DNA damage response (DDR) pathway, were increased in the *Pdgfra-Cre:Lmna*^F/F^ mouse hearts. Moreover, expression of senescence-associated beta-galactosidase was induced and levels of the senescence-associated secretory phenotype (SASP) proteins TGFβ1, CTGF (CCN2), and LGLAS3 were increased as well as the transcript levels of additional genes encoding SASP proteins in the *Pdgfra-Cre:Lmna*^F/F^ mouse hearts. Finally, expression of pH2AFX, a bonafide marker of the double-stranded DNA breaks, was increased in cardiac fibroblasts isolated from the *Pdgfra-Cre:Lmna*^F/F^ mouse hearts.

**Conclusion:**

Deletion of the *Lmna* gene in fibroblasts partially recapitulates the phenotype of the LMNA-associated DCM, likely through induction of double-stranded DNA breaks, activation of the DDR pathway, and induction of expression of the SASP proteins. The findings indicate that the phenotype in the LMNA-associated DCM is the aggregate consequence of the LMNA deficiency in multiple cardiac cells, including cardiac fibroblasts.

## One sentence summary

Cardiac fibroblasts contribute to the pathogenesis of DCM - associated with LMNA deficiency through activation of the senescence-associated secretory phenotype.

## INTRODUCTION

Heart failure is a major cause of mortality and morbidity in the world, affecting over 60 million people globally^[1,2]^. Hereditary cardiomyopathies are major causes of heart failure and sudden cardiac death^[1,3]^. Hereditary dilated cardiomyopathy (DCM) comprises a genetically heterogeneous group of disorders characterized by cardiac dilatation and systolic dysfunction^[4]^. Mutations in the *LMNA* gene, encoding nuclear envelope protein Lamin A/C (LMNA), are important causes of hereditary DCM^[4-7]^.

LMNA is expressed ubiquitously in almost all differentiated cells, including cardiac myocytes and fibroblasts^[8]^. Mutations in the *LMNA* gene cause a diverse array of clinical phenotypes, in addition to DCM, which are collectively referred to as laminopathies^[9,10]^. In accord with the ubiquitous expression of the *LMNA* gene in various cell types, the phenotypes in laminopathies are the aggregate consequences of the expression of the causal mutations in multiple cell types, tissues, and organs.

Cardiac involvement in laminopathies is common and typically manifests with DCM, atrial and ventricular conduction defects, supraventricular and ventricular arrhythmias, and sudden cardiac death^[6,7]^. DCM is the major cause of morbidity and mortality in laminopathies, particularly in the subset that predominantly involves the striated muscles^[5-7,11-13]^. *LMNA* mutations have been identified in up to 10% of the patients with familial DCM^[6,7]^. There is no specific therapy for laminopathies, including DCM- associated with the *LMNA* mutations.

The heart is a cellularly heterogeneous organ. Cardiac myocytes are considered the main cell type involved in the LMNA-associated DCM, a notion that is in accord with cardiomyopathies being considered mainly the diseases of the cardiac myocytes. Consequently, the contributions of other cardiac cell types, such as cardiac fibroblasts that also express the mutant LMNA to the pathogenesis of DCM, have remained unexplored. The purpose of the present study was to delineate the contribution of cardiac fibroblasts to the pathogenesis of DCM observed in laminopathies. Therefore, the *Lmna* gene was deleted in fibroblasts in mice upon expression of the Cre recombinase under the transcriptional controls of the platelet-derived growth factor receptor-α (*Pdgfra*) locus, which tags most of the fibroblasts in the heart. The ensuing cardiac phenotype was characterized in the *Lmna* heterozygous and homozygous mice.

## METHODS

### Data sharing and availability:

RNA-Seq data have been deposited in the public database GEO (Available online: https://www.ncbi.nlm.nih.gov/gds, GSE199078). Additional data about the present article are available from the corresponding author upon request.

### Regulatory approvals:

Animal procedures were in full compliance with the NIH Guidelines for the Care and Use of Laboratory Animals and approved by the Animal Care and Use Committee of the University of Texas Health Science Center - Houston (AWC-21-0015).

### Anesthesia and euthanasia:

Anesthesia was induced upon inhalation of 3% isoflurane and was maintained by inhalation of 0.5%-1% isoflurane throughout the procedures. Mice were euthanized with CO_2_ gas inhalation followed by cervical dislocation.

### Deletion of the *Lmna* gene in fibroblasts:

To delete the *Lmna* gene in fibroblasts, mice carrying floxed exon 2 of the *Lmna* gene (*Lmna*^F/F^) were crossed to the *Pdgfrα-Cre* BAC transgenic mice (JAX stock No: 013148)^[14,15]^. PDGFRA marks most of the cardiac fibroblasts, as indicated by its co-expression with collagen 1 (COL1A1) in more than 2/3rd of the isolated cardiac fibroblasts^[16]^. The *Pdgfrα-Cre:Lmna*^F/F^ and *Pdgfrα-Cre:Lmna*^W/F^ mice were generated along with the littermates that carried only the wild-type alleles (WT). The experimental groups included the WT, *Pdgfrα-Cre, Pdgfrα-Cre:Lmna*^W/F^, *Pdgfrα-Cre:Lmna*^F/F^ mice, which were matched for age and sex as properly applicable. Mice were housed in a 12-hours light/night cycle facility with food and water available *ad libitum*. DNA extracted from the tail clip was used for genotyping by PCR. The list of the oligonucleotide primers used for genotyping is presented in Supplementary Table 1.

### Survival:

Kaplan-Meier survival plots for the WT, *Pdgfrα-Cre, Pdgfrα-Cre:Lmna*^W/F^, and *Pdgfrα-Cre:Lmna*^F/F^ mice were constructed, and the survival rates were compared by the log-rank test.

### Bodyweight:

Body weight was measured weekly after two weeks of age in each mouse and compared among the WT, *Pdgfrα-Cre, Pdgfrα-Cre:Lmna*^W/F^, and *Pdgfrα-Cre:Lmna*^F/F^ mice.

### Metabolic panel:

Blood was collected from the retroorbital vein for the assessment of plasma metabolic panels, comprised of indices of kidney and liver functions as well as electrolytes, glucose, and lipid levels, in mice kept fast overnight.

### Echocardiography:

Cardiac size and function were assessed in age- and sex-matched mice in each of the four experimental groups by 2D, M mode, and Doppler echocardiography, as published using a Vevo 1100 ultrasound imaging system equipped with a 22-55 MHz MicroScan transducer (MS550D) (FUJIFILM VisualSonics Inc., Toronto, ON, Canada)^[17-19]^.

Echocardiography was performed in six weeks old WT, *Pdgfrα-Cre, Pdgfrα-Cre:Lmna*^W/F^, and *Pdgfrα-Cre:Lmna*^F/F^ mice. The selection of the time point was guided by the survival data, which enabled avoiding the potential confounding effects of the survival bias, as there was only sporadic death before six weeks of age. Echocardiography was also performed in 12 to 15 months old WT, *Pdgfrα-Cre*, and *Pdgfrα-Cre:Lmna*^W/F^ mice, given the late onset of the phenotype in the latter group. Finally, to identify the differentially expressed genes, before the onset of cardiac dysfunction, echocardiography was also performed in a subset of 3 to 4-week-old WT and *Pdgfrα-Cre:Lmna*^F/F^ mice, which were used for the isolation of cardiac fibroblasts and RNA-sequencing (RNA-Seq).

In brief, echocardiography was performed in mice lightly anesthetized with 0.5%-1% isoflurane inhalation. B-modes-guided M modes parasternal short-axis images were obtained in the supine position at the level of the tip of the papillary muscles. Left ventricular anterior wall thickness (LVAWT), LV posterior wall thickness (LVPWT), LV end-diastolic diameter (LVEDD), and LV end-systolic diameter (LVESD) were measured in 5 to 6 cardiac cycles using the leading-edge method, and the mean value of the measurements was calculated. LV fractional shortening and LV mass were calculated from the measured indices^[20]^. Echocardiographic measurements were indexed to body weight, as body weight is a strong determinant of indices of cardiac size, such as LVEDD, LVESD, and LVM. The corrected indices were defined as LVEDDI, LVESDI, and LVMI. Image acquisition and data analysis were performed without knowledge of the mouse genotype.

### Electrocardiography (ECG):

Two-lead precordial surface ECG was recorded by inserting subcutaneous needle electrodes into the upper right and left precordial area. During the recording, mice were kept lightly anesthetized using 0.5%-1% isoflurane and under constant monitoring on a 37 °C heated pad. Leads were connected to a PowerLab 4/30 System using an Animal Bio Amp module (ADInstruments, Colorado Springs, CO, USA). The rhythm was recorded and visualized using LabChart7 Software without knowledge of the genotype.

### Myocyte cross-sectional area (CSA):

To determine cardiac myocyte CSA, thin myocardial sections were stained with wheat germ agglutinin (WGA) to mark the extracellular matrix and myocyte boundaries, as published^[17,18]^. The total area unstained with WGA was determined using the Image J software (Available online: https://imagej.net) and used to calculate the myocyte CSA. Approximately 20,000 cells per mouse and 4 to 5 mice per genotype were analyzed.

### Myocardial Fibrosis:

Myocardial fibrosis was detected upon staining of thin myocardial sections with picrosirius red, as published^[17,18]^. Collagen volume fraction (CVF) was calculated as the percent area of the myocardial section stained positive for picrosirius red using the Image J software in 10 high magnification microscopic fields (×40) per section, in 6 sections per heart, and a minimum of 4 mice per each experimental group, as published^[17,18]^.

### Myocardial adipocytes:

To detect adipocytes in the heart, thin myocardial sections were prepared from the paraffin-embedded tissues and subjected to antigen retrieval upon treatment with 10 mM of boiling sodium citrate (pH 6.0). The sections were incubated overnight with an anti-perilipin 1 (PLIN1) antibody to identify lipid-containing adipocytes (Cell Signaling Technology, cat. # 9349), as published^[19]^. Further, sections were washed with PBS, incubated with the secondary antibody conjugated to Alexa 594, and counterstained with 4′,6 Diamidino-2-phenylindole dihydrochloride (DAPI, Sigma-Aldrich St Louis, MO; cat. # D8417) at 1 μg/mL concentration to mark the nuclei. After mounting the sections in fluorescence mounting media (DAKO, cat. # S3023), they were imaged using a Zeiss Axioplan fluorescence microscope. The number of PLIN1-stained adipocytes was counted in 10 high magnification fields (×40) per section and 6 sections per heart, and at least 4 hearts per genotype. The mean numbers of the adipocytes per section were compared among the groups.

### TUNEL assay:

Cells undergoing apoptosis in the myocardium were detected using the terminal deoxynucleotidyl transferase dUTP nick end labeling (TUNEL) assay and the In-Situ Cell Death Detection Fluorescein Kit, as published^[18,21]^. The number of cells stained positive for the TUNEL assay was determined in approximately, 10,000 cells per heart and in 5 to 6 hearts per genotype, and the percentages were compared.

### Isolation of cardiac fibroblasts:

Mice were anesthetized with an intraperitoneal injection of pentobarbital (62 mg/kg, ip) and anti-coagulated upon IP administration of 200 units of heparin. The heart was harvested, rinsed in a cold PBS buffer, and cannulated through the ascending aorta. The cannula was positioned above the aortic valves and connected to a retrograde perfusion system. The heart was perfused with PBS at a constant rate of 4 mL/min until blood was fully rinsed. Then, collagenase II (Worthington, Lakewood, NJ) was added to the perfusion buffer at 25 mg/25 mL of 250 units/mL solution, and the heart was perfused until complete softening of the myocardium (typically for about 8 minutes). The heart was then minced into small pieces in the collagenase buffer and cells were dissociated by gentle pipetting. Upon complete dissociation of the tissue, the collagenase activity was stopped by adding calf serum (10% in the final volume), and the cell suspension was passed through a 40 μm mesh cell strainer to remove the debris. The cells were then centrifuged at 300 *g* for 10 min at 4 °C, and the pellet was resuspended in a MACS buffer (Miltenyi Biotec, Bergisch Gladbach, Germany; cat. # 130-091-221) and washed with PBS. The cells were then incubated with an antibody against PDGFRA (cat. # 17-1401-81, eBioscience) and a cocktail of mouse lineage antibodies (cat. # 561301, BD Pharmingen, BD Horizon™ V450 Mouse Lineage Antibody Cocktail, with Isotype Control) for 1 h at 4 °C in the dark. Following antibody treatment, 4′,6 Diamidino-2-phenylindole dihydrochloride (DAPI, cat. # D8417, Sigma-Aldrich) was added at 0.1 mg/mL to the cell suspension for 10 minutes. The unbound antibodies and excess DAPI were removed by washing the cells twice in the MACS buffer. The cell suspension was passed through a mesh strainer before sorting through a FACS-Aria flow cytometer (BD Pharmingen, San Diego, CA). Cells were sorted to isolate living (DAPI^neg^) and hematopoietic lineage negative (Lin^neg^) cells that expressed PDGFRA (PDGFRA^pos^ cells). Unlabeled cells, as well as cells stained with the appropriate isotype IgG controls (cat. # 17-4321, eBioscience, APC Rat IgG2a K Isotype Control), were included as controls.

### Cardiac myocyte isolation:

Cardiac myocytes were isolated as published^[17-19,22]^. In brief, the heart was excised from the anesthetized mice and perfused retrogradely with the digestion collagenase buffer at a concentration of 2.4 mg/mL concentration at a flow rate of 4 mL/min (Worthington cat. # LS004176). Following the enzymatic digestion, the heart was minced in a 10% calf serum, 12.5 μM CaCl_2_, and 2 mM ATP buffer, and the cell suspension was filtered through a 100 μm cell strainer. Cardiac myocytes per precipitated by centrifugation at 20 *g* and re-introduction of an increasing concentration of calcium in a stepwise fashion with a final concentration of 900 μM added to the stop buffer. Then, the cardiac myocytes were suspended in a Qiazol reagent (Qiagen cat. # 79306) for RNA extraction, in a protein extraction buffer for immunoblotting, or fresh frozen in isopentane for immunofluorescence studies.

### Immunoblotting.

Total protein was extracted from cardiac fibroblasts, quantified by the Bradford assay, and was used for immunoblotting, as published^[18,21,23]^. Approximately 50 μg aliquots of the protein extracts were loaded onto polyacrylamide gels, separated by electrophoresis, transferred to a nitrocellulose membrane, and probed with the desired antibody. The primary and secondary antibodies used are listed in Supplementary Table 1.

### Immunofluorescence:

To detect the expression and localization of the protein of interest, thin myocardial frozen sections were prepared and probed with specific primary antibodies as published^[19]^. The antibodies used for the immunofluorescence studies are listed in Supplementary Table 1.

### Senescence β-galactosidase staining:

Senescence β-galactosidase staining kit (Cell Signaling Technology, cat. # 9860) was used to detect β-galactosidase activity according to the manufacturer’s protocol. Briefly, thin fresh frozen myocardial sections were fixed using the 1X fixative solution for 15 min and washed twice with 1X PBS. The fixed sections were incubated with the β-galactosidase solution in a sealed humid box overnight at 37 °C in a dry incubator (no CO_2_). The sections were rinsed with PBS, counterstained with Eosin, and mounted using 70% glycerol for long-term storage. The images were taken using a 2× magnification using an Olympus BX40 microscope.

### RT-PCR:

To detect and compare transcript levels of the selected genes, RT-PCR was performed as published^[17,19]^. In brief, total RNA was extracted using the miRNeasy Mini Kit (cat. # 217004, Qaigen Inc.), treated with DNase, and reverse transcribed into cDNA using the High-Capacity cDNA Reverse Transcription Kit (Life Tech, cat. # 4368814). The RT-PCR was performed using the TaqMan gene expression assays or SYBR Green specific primers. Glyceraldehyde-3-phosphate dehydrogenase (Gapdh) mRNA levels were used as loading controls for normalization. Gene expression was compared using the ΔCT method and the data were presented as normalized levels. The TaqMan probes and SYBR Green primers are listed in Supplementary Table 1.

### Sequencing (RNA-Seq):

RNA-Seq was performed on ribosome-depleted RNA isolated from cardiac fibroblasts, as published, with some modifications^[18,21,23]^. In brief, cardiac fibroblasts were isolated from the ventricular tissues and used for total RNA extraction. The RNA samples that had an RNA Integrity Number (RIN) of > 8 were used to generate strand-specific RNA sequencing libraries. The sequencing reaction was performed on an Illumina instrument, and 150 bp paired-end reads were generated and aligned to the mouse reference genome build mm10 using HISAT2^[24]^. The GENCODE program (Available online: https://www.gencodegenes.org/mouse/) was used to annotate the aligned read pairs. Read counts were determined using the featureCounts upon RUV normalization, and the differentially expressed genes (DEGs) were identified using the DESeq2 program in the R package (Available online: https://www.r-project.org/)^[25,26]^. A *Q*-value (Benjamini-Hochberg FDR-adjusted *P*-value) of < 0.05 was used to define the DEGs^[27]^.

### Pathway analysis:

The RNA-seq data were analyzed using GSEA to identify gene sets that were enriched in the RNA-Seq data, as compared to the curated gene sets for Hallmark and the canonical pathways of the Molecular signature database (MSigDB). An FDR of < 0.05 was used to identify significantly enriched pathways.

To identify transcriptional regulators (TRs) that were predicted to be activated or suppressed, the DEGs were overlapped with the curated genes in each transcriptional pathway using the Ingenuity pathway analysis (IPA) software. The predicated TRs were defined based on a *P*-value of < 0.05 and a *Z* score of greater than +2 or less than −2.

### Statistical analysis:

Data were analyzed for a Gaussian distribution using the Shapiro-Wilk normality test. Data following the Gaussian distribution were compared by the *t*-test or one-way ANOVA followed by Bonferroni pairwise comparisons. The data that deviated from a Gaussian distribution and the categorical data were analyzed by the Kruskal-Wallis and the Chi-Square test, respectively. Survival rates were analyzed by the Log-rank test. Statistical analyses were performed using Graph pad Prism 8 or STAT IC, 15.1.

## RESULTS

### The efficiency of deletion of the *Lmna* gene in cardiac fibroblasts:

**Figure 1.**
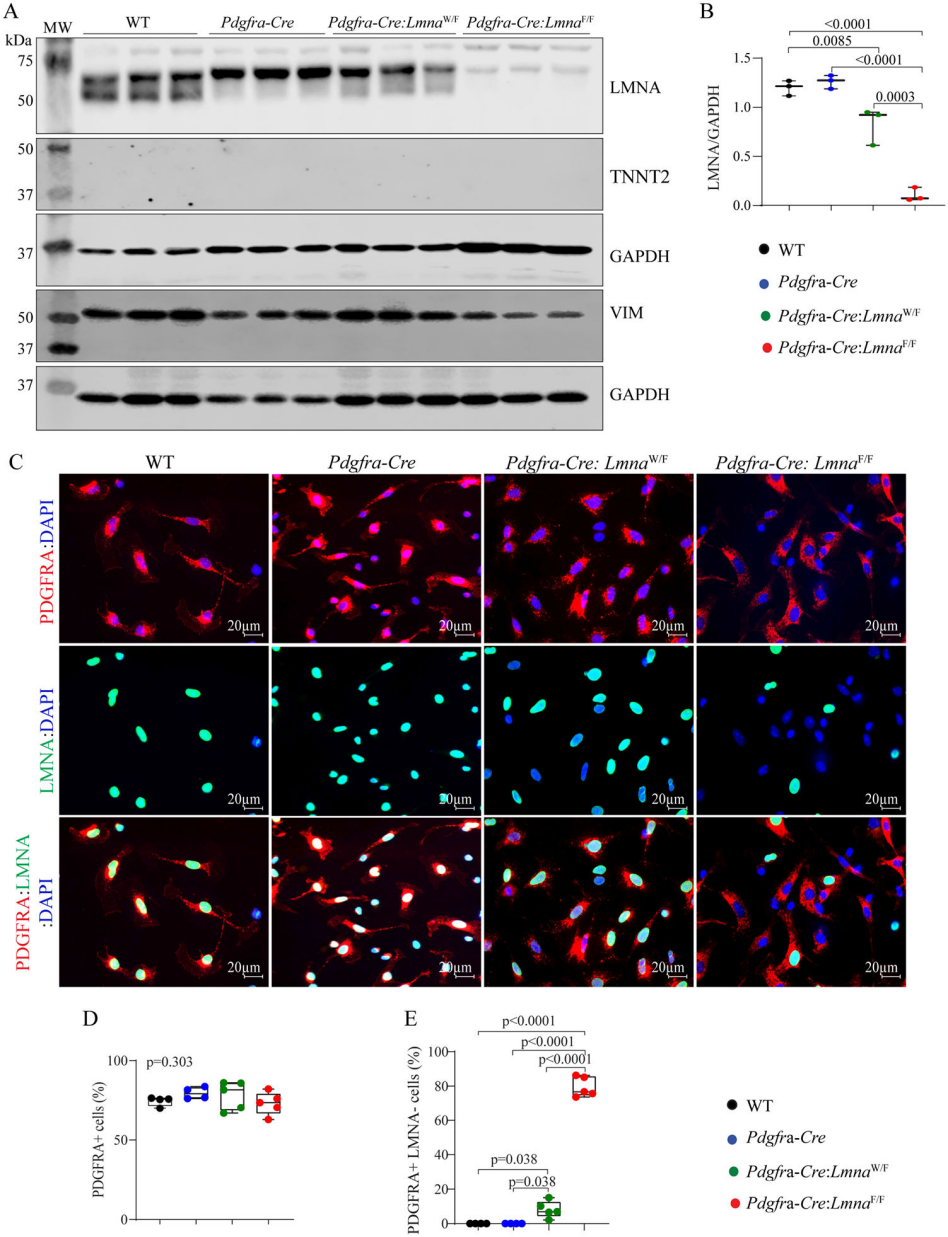
Fidelity of conditional deletion of the *Lmna* gene in cardiac fibroblasts. (A) Immunoblots showing LMNA protein levels in cardiac fibroblasts isolated from the wild type (WT), *Pdgfra-Cre*, *Pdgfra-Cre:Lmna*^W/F^, and *Pdgfra-Cre:Lmna*^F/F^ mice. The upper panel shows the LMNA protein. Blots representing cardiac troponin T (TNNT2) and vimentin (VIM), as markers of cardiac myocytes and fibroblasts, are included to assess the fidelity of the cell isolation. Blots representing GAPDH are included as loading controls. (B) Quantitative data on the LMNA protein levels corresponding to the blots in panel A. (C) Immunofluorescence staining of isolated cardiac fibroblasts for the expression of PDGFRA (upper panels) and LMNA (middle panel) and the overlay (lower panel). (D) Panel D the percentages of the isolated non-myocyte cells expressing PDGFRA. (E) Percentage of the PDGFRA expressing cells in which the LMNA protein was absent. In all analyses in the present and subsequent Figures, pairwise *P*-values were presented only when the *P*-value by the one-way ANOVA test was significant.

To determine the efficiency of deletion of the *Lmna* gene, cardiac fibroblasts were isolated from the WT, *Pdgfra*-Cre, *Pdgfra-Cre:Lmna*^W/F^, and *Pdgfra-Cre:Lmna*^F/F^ mouse hearts and the protein extracts from these cells were probed with an anti-LMNA antibody. LMNA protein levels were reduced by about 89.21 ± 5.62 in the cardiac fibroblast isolated from the *Pdgfra-Cre:Lmna*^F/F^ and 17.12 ± 15.23 in the *Pdgfra-Cre:Lmna*^W/F^ mice as compared to the WT or *Pdgfra-Cre* mice [Figure 1A and B]. To exclude cross-contamination of the fibroblast fraction with other cardiac cell types and verify the authenticity of the protein isolates, the membranes were probed with the antibodies against cardiac troponin T (TNNT2), representing cardiac myocytes, and vimentin (VIM), representing fibroblasts. Whereas expression of TNNT2 was not detected by immunoblotting in the protein isolates from cardiac fibroblasts, VIM was abundantly expressed in the protein extracts from cardiac fibroblasts [Figure 1A].

To complement the findings, isolated cardiac fibroblasts were also co-immunostained with antibodies against LMNA and PDGFRA, as well as DAPI, the latter to stain the nuclei. Approximately 73.1% ±7.1% to 79.6% ± 3.7% of the isolated non-myocyte cardiac cells expressed PDGFRA showing no difference among the genotypes, as detected by immunofluorescence staining [Figure 1C and D]. All isolated non-myocyte cardiac cells expressing PDGFRA also expressed LMNA in the WT and *Pdgfra-Cre* groups, consistent with the ubiquitous expression of the LMNA in all post-natal cells [Figure 1C and E]. Expression of the LMNA was absent in 8.2% ± 4.8% of the non-myocyte cells expressing PDGFRA in the *Pdgfra-Cre:Lmna*^W/F^ mice, which is reflective of one copy of the *Lmna* gene being intact and expressing the LMNA protein. The LMNA expression was not detected in 79.5% ± 5.8% of the non-myocyte cells that stained positive for the PDGFRA in the *Pdgfra-Cre:Lmna*^F/F^ mice, indicating effective ablation of both copies of the *Lmna* gene in the isolated cardiac fibroblasts in the latter genotype [Figure 1C and E].

**Figure 2.**
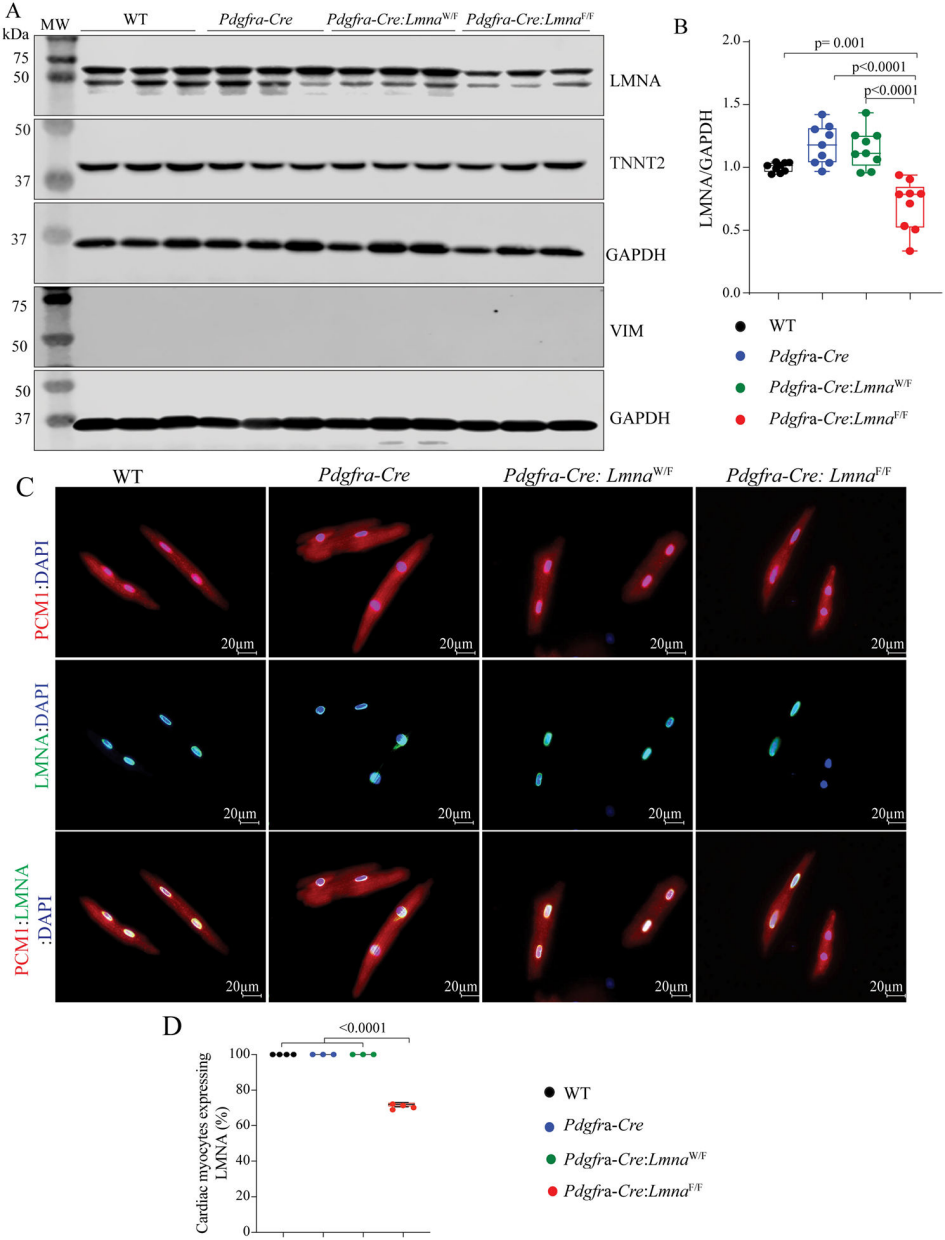
Fortuitous deletion of the *Lmna* gene in a subset of cardiac myocytes. (A) Immunoblots showing LMNA protein levels in cardiac myocytes isolated the WT, *Pdgfra-Cre*, *Pdgfra-Cre:Lmna*^W/F^, and *Pdgfra-Cre:Lmna*^F/F^ mice. As in Figure 1, blots representing TNNT2 and VIM are included to assess the fidelity of the cell isolation. Blots representing GAPDH are included as controls for the loading conditions. (B) Quantitative data corresponding to the blots shown in F, showing about 30% reduction in the LMNA protein levels in the cardiac myocytes isolated from the *Pdgfra-Cre:Lmna*^F/F^ mice, whereas the LMNA levels were unchanged in the myocytes isolated from the *Pdgfra-Cre* and *Pdgfra-Cre:Lmna*^W/F^ mice. (C) Immunofluorescence staining of isolated cardiac myocytes for the PCM1 expression (upper panel), which tags cardiac myocyte nuclei in the heart, LMNA (middle panel), and the overlay (lower panel). (D) Quantitative data showing the percentages of cardiac myocytes, identified by the expression of PCM1, also express the LMNA proteins.

The *Pdgfra* locus is transcriptionally active in a small subset of cardiac myocytes during embryonic development but not in the adult heart^[16]^. To determine whether deletion of the *Lmna* gene under the transcriptional control of the *Pdgfra* locus (*Pdgfra-Cre* mice) also affects the expression of the LMNA protein in cardiac myocytes, protein extracts from adult mouse cardiac myocytes were probed for the expression of the LMNA protein by immunoblotting and immunofluorescence staining. The LMNA protein levels were unchanged in the *Pdgfra-Cre:Lmna*^W/F^ mouse myocytes but were reduced by ~30% in the *Pdgfra-Cre:Lmna*^F/F^ mouse myocytes [Figure 2A and B]. To assess the purity of the isolates, the membranes were probed with antibodies against TNNT2 and VIM, representing cardiac myocytes and fibroblasts, respectively. As would be expected, TNNT2 but not VIM was expressed in the cardiac myocyte protein isolates, as detected by immunoblotting [Figure 2A]. Immunofluorescence staining of isolated cardiac myocytes identified the absence of the LMNA protein in 29.28% ± 1.18% of cardiac myocytes [Figure 2C and D].

### Growth and survival:

**Figure 3.**
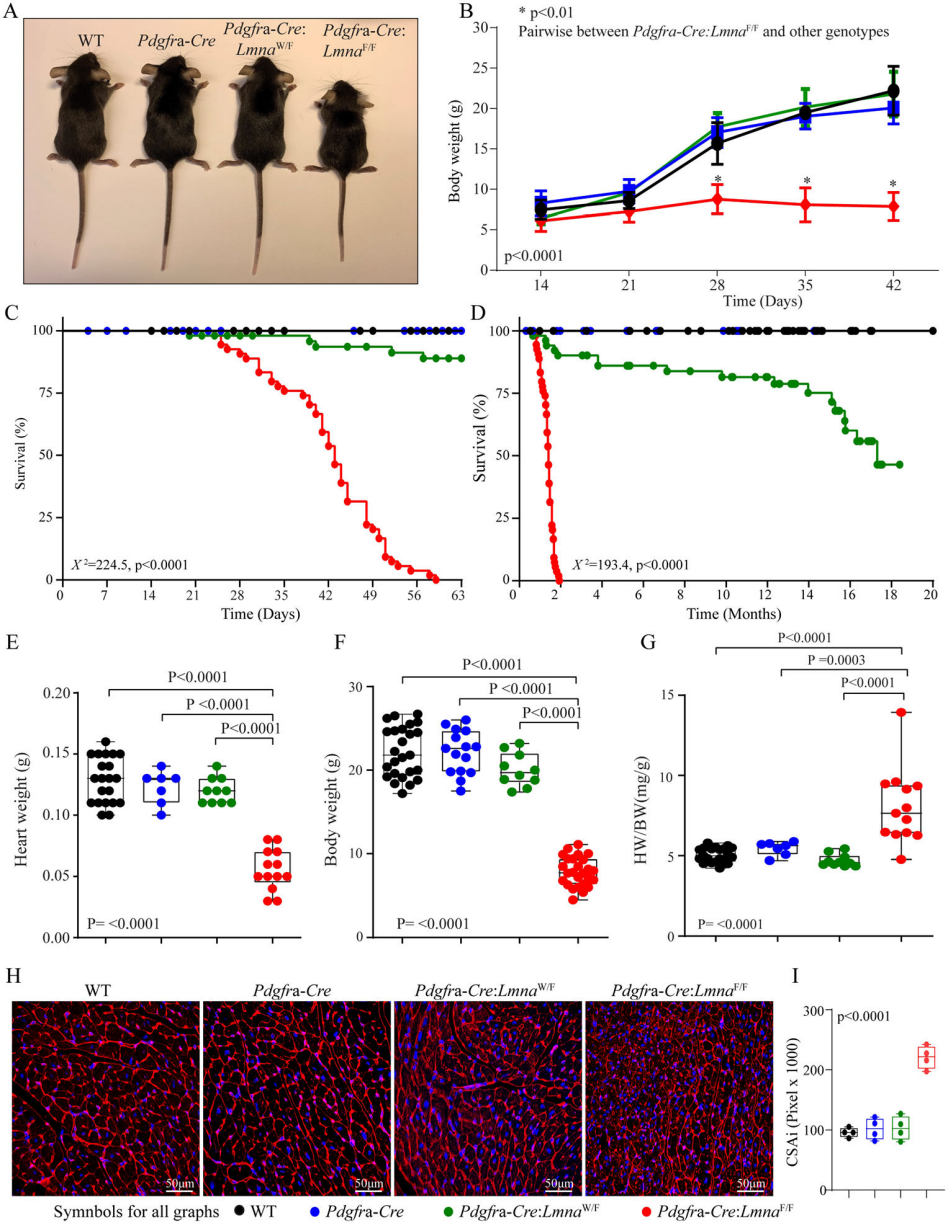
Gross phenotype and survival. (A) Gross morphology of the WT, *Pdgfra-Cre*, *Pdgfra-Cre:Lmna*^W/F,^ and *Pdgfra-Cre:Lmna*^F/F^ mice at six weeks of age. (B) Growth curves of the mice in the experimental groups up to six weeks of age. (C) Kaplan-Meier survival plots show the survival rates up to ~2 months of age, the longest survival time of the *Pdgfra-Cre:Lmna*^F/F^ mice. (D) Kaplan-Meier survival plots show the survival rates up to 20 months of age. Log-rank test p-value is shown. (E-G) Heart weight (E), body weight (F), and heart weight to body weight ratio (HW/BW) of the WT, *Pdgfra-Cre*, *Pdgfra-Cre:Lmna*^W/F^, and *Pdgfra-Cre:Lmna*^F/F^ mice at six weeks of age. (H) Wheat Germ Agglutinin (WGA) stained thin myocardial sections used to calculate myocyte cross-sectional area (CSA). I. Graph representing myocyte CSA indexed to heart weight (CSAI) in the control and experimental groups.

The *Pdgfra-Cre:Lmna*^F/F^ mice exhibited growth retardation as evidenced by smaller body weight as compared to the WT and *Pdgfra-Cre* mice, whereas the *Pdgfra-Cre:Lmna*^W/F^ mice had a normal growth rate [Figure 3A and B]. Likewise, the *Pdgfra-Cre:Lmna*^F/F^ mice exhibited increased mortality starting at around 3-4 weeks of age and reaching 100% mortality by two months of age [Figure 3C]. The *Pdgfra-Cre:Lmna*^W/F^ mice also showed increased mortality, albeit later in life and reaching mortality of ~50% at 18 months of age [Figure 3D], whereas the WT and *Pdgfra-Cre* mice had normal survival rates.

### Metabolic panel:

Plasma levels of electrolytes, glucose, and indices of kidney and liver function were comparable among the WT, *Pdgfra-Cre:Lmna*^W/F^ and *Pdgfra-Cre:Lmna*^F/F^ mice, except for plasma levels of glucose, which was reduced in the *Pdgfra-Cre:Lmna*^F/F^ mice [Supplementary Table 2].

### Cardiac size and function:

The heart weight was smaller in the *Pdgfra-Cre:Lmna*^F/F^ mice, reflective of the smaller body size, while the heart weight/body weight ratio was increased in the *Pdgfra-Cre:Lmna*^F/F^ mice [Figure 3E-G]. There were no differences in the heart weight or heart weight/body weight ratio among the WT, *Pdgfra-Cre*, and *Pdgfra-Cre:Lmna*^W/F^ mice [Figure 3E-G]. To determine cardiac myocyte size, thin myocardial sections were stained with WGA, and cardiac myocyte CSA was calculated and indexed to heart weight as published^[22,28]^. Cardiac myocyte CSA was increased in the *Pdgfra-Cre:Lmna*^F/F^ mice [Figure 3H and I].

LV size and function were assessed by echocardiography at six weeks of age in all four groups and 12-18 months of age in the WT, *Pdgfra-Cre*, and *Pdgfra-Cre:Lmna*^W/F^ mice. The time point of six weeks was selected based on the survival data showing increased mortality after six weeks of age to avoid survival bias. At six weeks of age, the LVEDDI, LVESDI, and LVMI were increased and LVFS was markedly reduced in the *Pdgfra-Cre:Lmna*^F/F^ mice, as compared to the WT or *Pdgfra-Cre* mice [Table 1]. The *Pdgfra-Cre:Lmna*^W/F^ mice exhibited normal echocardiographic indices of cardiac size and function at six weeks of age, but when assessed at 12 to 18 months of age, the heterozygous mice exhibited LV dilatation and dysfunction, as indicated by increased LVESDI and LVFS, as compared to WT or *Pdgfra-Cre* mice [Table 2]. Likewise, LVMI was significantly increased in the *Pdgfra-Cre:Lmna*^W/F^ mice [Table 2].

### Cardiac arrhythmias:

**Figure 4.**
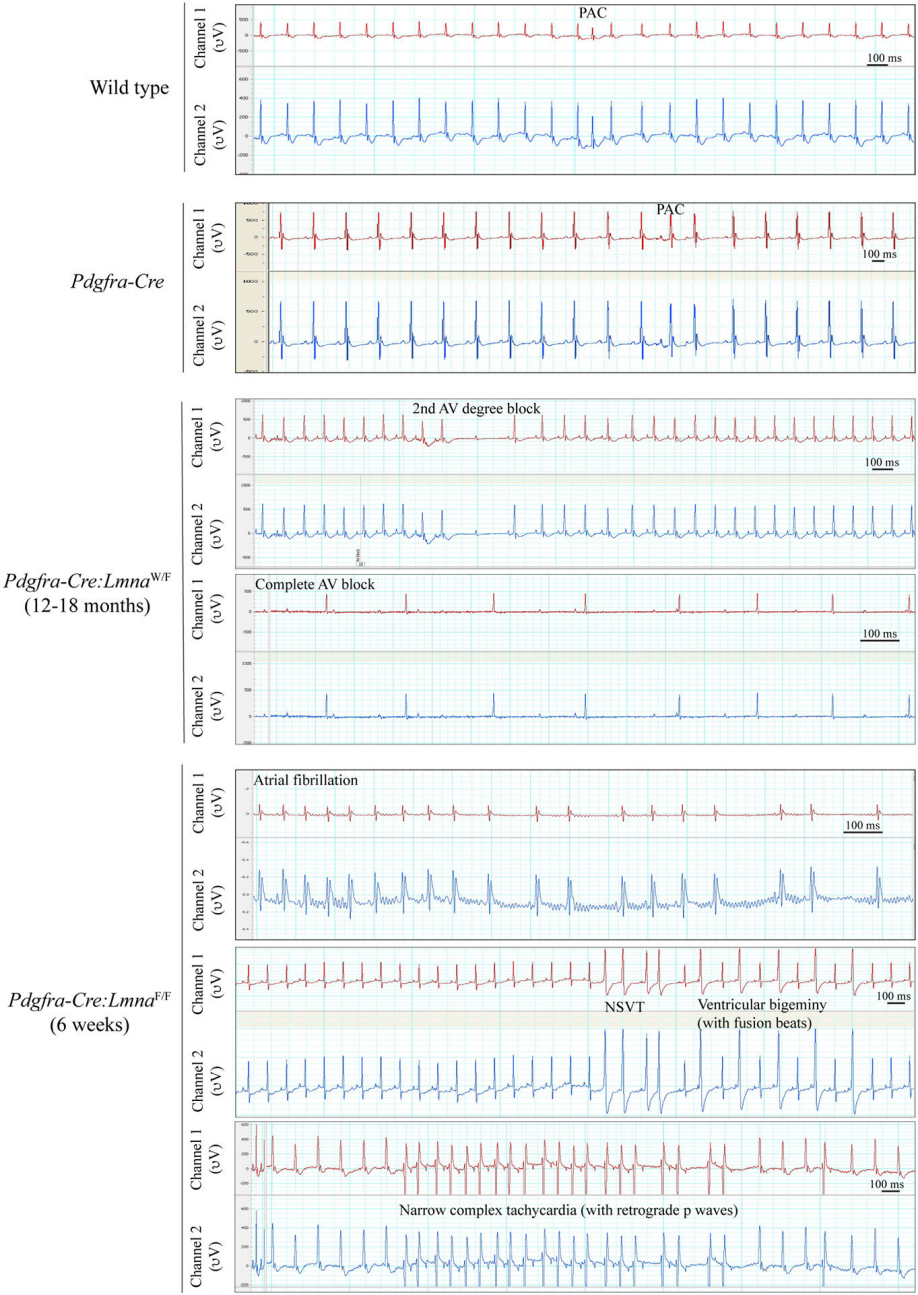
Electrocardiographic recordings. Two-channel cardiac rhythm recordings are shown in the WT, *Pdgfra-Cre*, *Pdgfra-Cre:Lmna*^W/F^, and *Pdgfra-Cre:Lmna*^F/F^ mice. The *Pdgfra-Cre:Lmna*^W/F^ mice showed second and third-degree atrioventricular blocks after one year of age (third set of panels). The *Pdgfra-Cre:Lmna*^F/F^ mice exhibited runs of wide complex and narrow complex tachycardia episodes at 6 weeks of age (the lower panels).

Cardiac rhythm, monitored for about an hour in each mouse (8-12 mice per group), was notable for the presence of supraventricular and ventricular tachycardia as well as atrioventricular (AV) blocks in the Lmna-deficient mice [Figure 4]. Specifically, 4/12 *Pdgfra-Cre:Lmna*^F/F^ mice, monitored at six weeks of age, developed atrial fibrillation, 3/12 had non-sustained ventricular tachycardia and 1/12 had a narrow QRS tachycardia with apparent retrograde p wave [Figure 4]. The *Pdgfra-Cre:Lmna*^W/F^ mice did not show notable arrhythmias at six weeks of age; however, they exhibited ventricular arrhythmias and AV blocks, including complete heart block at 12-18 months of age [Figure 4].

### Myocardial fibrosis:

**Figure 5.**
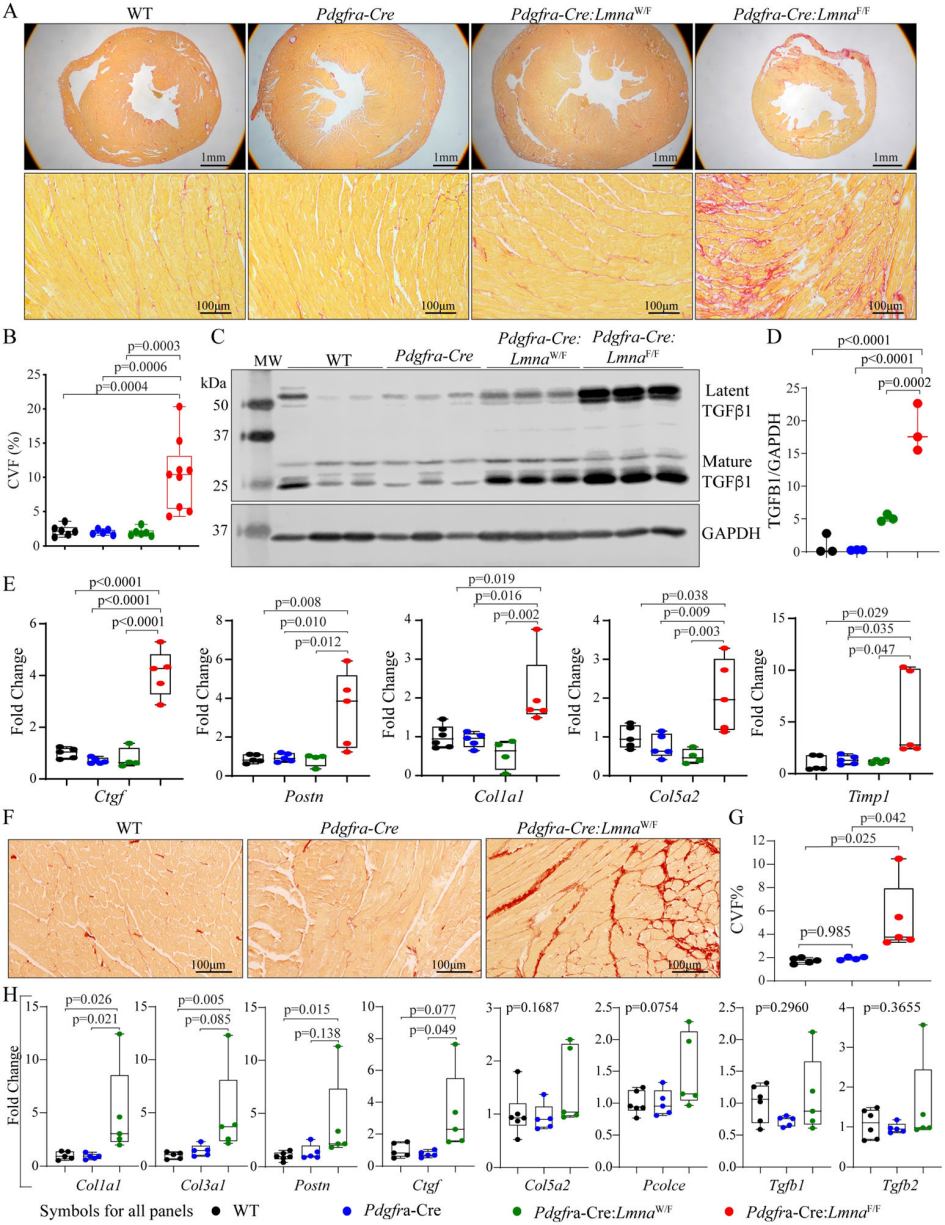
Myocardial fibrosis. (A) Representative images of Low (upper) and high (lower) magnification of thin myocardial sections stained for picrosirius red, which illustrates increased myocardial fibrosis in the *Pdgfra-Cre:Lmna*^F/F^ mice at six weeks of age. (B) Quantitative data showing collagen volume fraction (CVF) in the experimental and control groups. (C) Immunoblot showing levels of latent and mature TGFβ1 in the control and experimental groups, which were markedly increased in the *Pdgfra-Cre:Lmna*^W/F^ and *Pdgfra-Cre:Lmna*^F/F^ mice. (D) Quantitative data representing the blot in panel C. (E) Transcript levels of selected markers of myocardial fibrosis, quantified by RT-PCR, in the control and experimental groups. (F) Thin myocardial sections stained for picrosirius red in the *Pdgfra-Cre:Lmna*^W/F^ and control mice, showing increased myocardial fibrosis. (G) Quantitative data showing increased CVF in the *Pdgfra-Cre:Lmna*^W/F^ mouse myocardium as compared to the WT and *Pdgfra-Cre* mice at one year of age. (H) Transcript levels of selected markers of fibrosis, showing increased levels of selected markers but not Tgfb1 in the myocardium of one-year-old *Pdgfra-Cre:Lmna*^W/F^ mice.

CVF, calculated from picrosirius red-stained thin myocardial sections, comprised 2.2% ± 0.8%, 2.0% ± 0.4%, 2.0% ± 0.6%, and 10.4% ± 5.2% of the myocardium in the six-week-old WT, *Pdgfra-Cre*, *Pdgfra-Cre:Lmna*^W/F^, and *Pdgfra-Cre:Lmna*^F/F^ mice, respectively [Figure 5A and B]. In agreement with increased CVF, levels of latent and mature TGFβ1, a major pro-fibrotic mitotic factor, were markedly increased in the *Pdgfra-Cre:Lmna*^W/F^ and *Pdgfra-Cre:Lmna*^F/F^ mouse hearts [Figure 5C and D]. In addition, transcript levels of a selected number of genes involved in myocardial fibrosis, namely, *Ctgf* (*Ccn2*), *Postn*, *Col1a1*, *Col5a2*, and *Timp*, quantified by RT-PCR, were also increased in the *Pdgfra-Cre:Lmna*^F/F^ mouse hearts [Figure 5E].

CVF was also quantified in 12-18 months old *Pdgfra-Cre:Lmna*^W/F^ mice, which comprised 5.3% ± 3.0% of the myocardium, as opposed to 1.7 ± 0.2 and 1.9% ± 0.1% in the WT and *Pdgfra-Cre* mice, respectively [Figure 5D and E]. Similarly, transcript levels of *Col1a1*, *Col3a1*, *Postn*, and *Ctgf* (*Ccn2*) were increased in the *Pdgfra-Cre:Lmna*^W/F^ mouse hearts, while levels of *Colsa2*, *Pcolce*, *Tgfb1*, and *Tgfb2* were unchanged [Figure 5H].

### Myocardial apoptosis:

**Figure 6.**
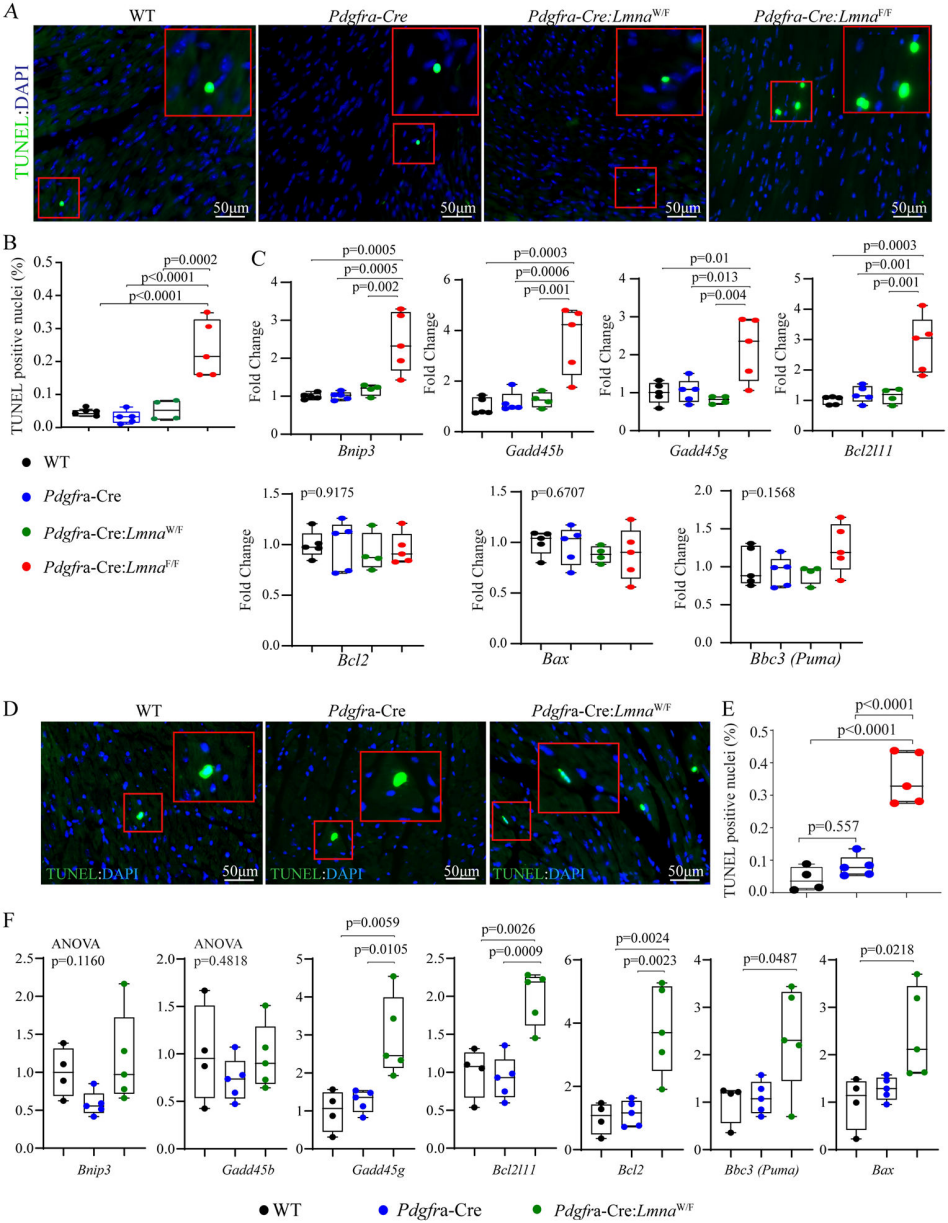
Myocardial apoptosis. (A) The three panels show thin myocardial sections stained for the TUNEL assay. The inserts show a high magnification view of the selected areas marked with a red box. The data are from six weeks old mice. (B) Quantitative data showing the percentages of the TUNEL positive cells in the experimental and control groups at six weeks of age. (C) Transcript levels of selected markers of apoptosis, quantified by RT-PCR, in the control and the experimental groups. (D) Thin myocardial sections were stained for the TUNEL assay in the one-year-old *Pdgfra-Cre:Lmna*^W/F^ and control mice. (E) Quantitative data showing an increased number of nuclei stained positive for the TUNEL assay in the one-year *Pdgfra-Cre:Lmna*^W/F^ mice, as compared to the WT and the *Pdgfra-Cre* mice. (F) Transcript levels of selected markers of apoptosis, showing increased levels of the selected genes in the one-year-old *Pdgfra-Cre:Lmna*^W/F^ mice, as compared to the control groups.

The number of myocardial cells undergoing apoptosis, detected by the TUNEL assay, was increased in the myocardium of *Pdgfra-Cre:Lmna*^F/F^ mice at six weeks of age, as compared to the WT, *Pdgfra-Cre*, and *Pdgfra-Cre:Lmna*^W/F^ mice [Figure 6A and B]. Likewise, transcript levels of several genes involved in apoptosis, namely *Bnip3*, *Gadd45b*, *Gadd45g*, and *Bcl2l11*, were increased in the myocardium of *Pdgfra-Cre:Lmna*^F/F^ mice, whereas those of *Bcl2*, *Bbc3*, and *Bax* were unchanged [Figure 6C]. Analysis of the *Pdgfra-Cre:Lmna*^W/F^ mice at an older age also showed an increased number of the myocardial cells that stained for the TUNEL assay as well as increased transcript levels of *Bax*, *Bbc3*, *Bcl2*, *Gadd45g*, and *Bcl2l11* but not *Bnip3* and *Gadd45b* [Figure 6D-F].

### Myocardial adipocytes:

**Figure 7.**
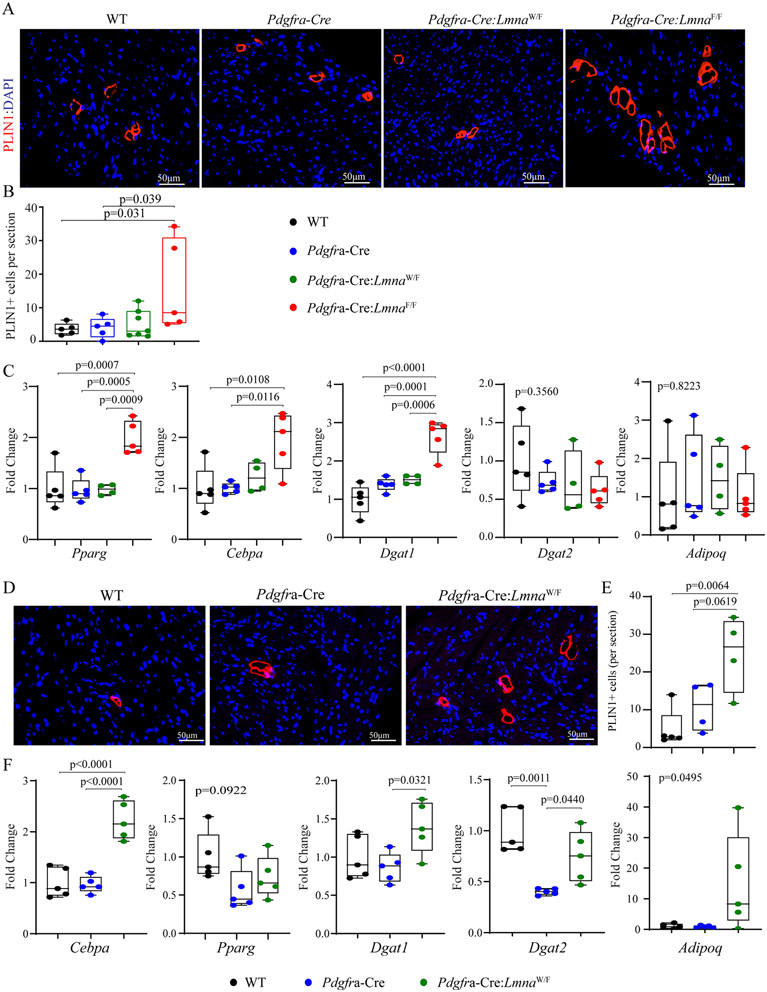
Myocardial adipocytes. (A) Thin myocardial sections were stained for the expression of perilipin 1 (PLIN1), a marker for triglyceride-rich adipocytes in 6 weeks old mice. (B) Quantitative data showing the number of cells stained positive for the expression of PLIN1 per myocardial section in the experimental and control groups at six weeks of age. (C) Transcript levels of selected markers of adipogenesis, quantified by RT-PCR, in the control and experimental groups. (D) Thin myocardial sections were stained for the expression of PLIN1 in the one-year-old *Pdgfra-Cre:Lmna*^W/F^ and the control mice. (E) Quantitative data showing the number of PLIN1 expressing cells per section in one-year-old WT, *Pdgfra-Cre*, and *Pdgfra-Cre:Lmna*^W/F^ mice. (F) Transcript levels of selected markers of adipogenesis in one-year-old WT, *Pdgfra-Cre*, and *Pdgfra-Cre:Lmna*^W/F^ mice.

To detect adipocytes in the myocardium, thin myocardial sections were stained with an antibody against perilipin 1 (PLIN1), which marks the triglyceride-laden fat cells. The number of adipocytes was increased in the *Pdgfra-Cre:Lmna*^F/F^ mouse hearts as compared to the WT, *Pdgfra-Cre*, and *Pdgfra-Cre:Lmna*^W/F^ mouse hearts [Figure 7A and B]. In addition, transcript levels of *Pparg*, *Cebpa*, and *Dgat1*, major regulators of adipogenesis, were increased in the *Pdgfra-Cre:Lmna*^F/F^ mouse hearts, whereas levels of *Dgat2*, and *Adipoq* transcripts were unchanged [Figure 7C]. The heterozygous *Lmna* mice were also analyzed for adipogenesis at age 12-18 months of age, which also showed an increased number of myocardial cells expressing PLIN1 as compared to the WT or *Pdgfra-Cre* mice [Figure 7D and E]. Likewise, levels of the *Cebpa*, a major regulator of adipogenesis, were markedly increased in the *Pdgfra-Cre:Lmna*^W/F^ mouse hearts [Figure 7F].

### DEGs in the LMNA-deficient cardiac fibroblasts:

**Figure 8.**
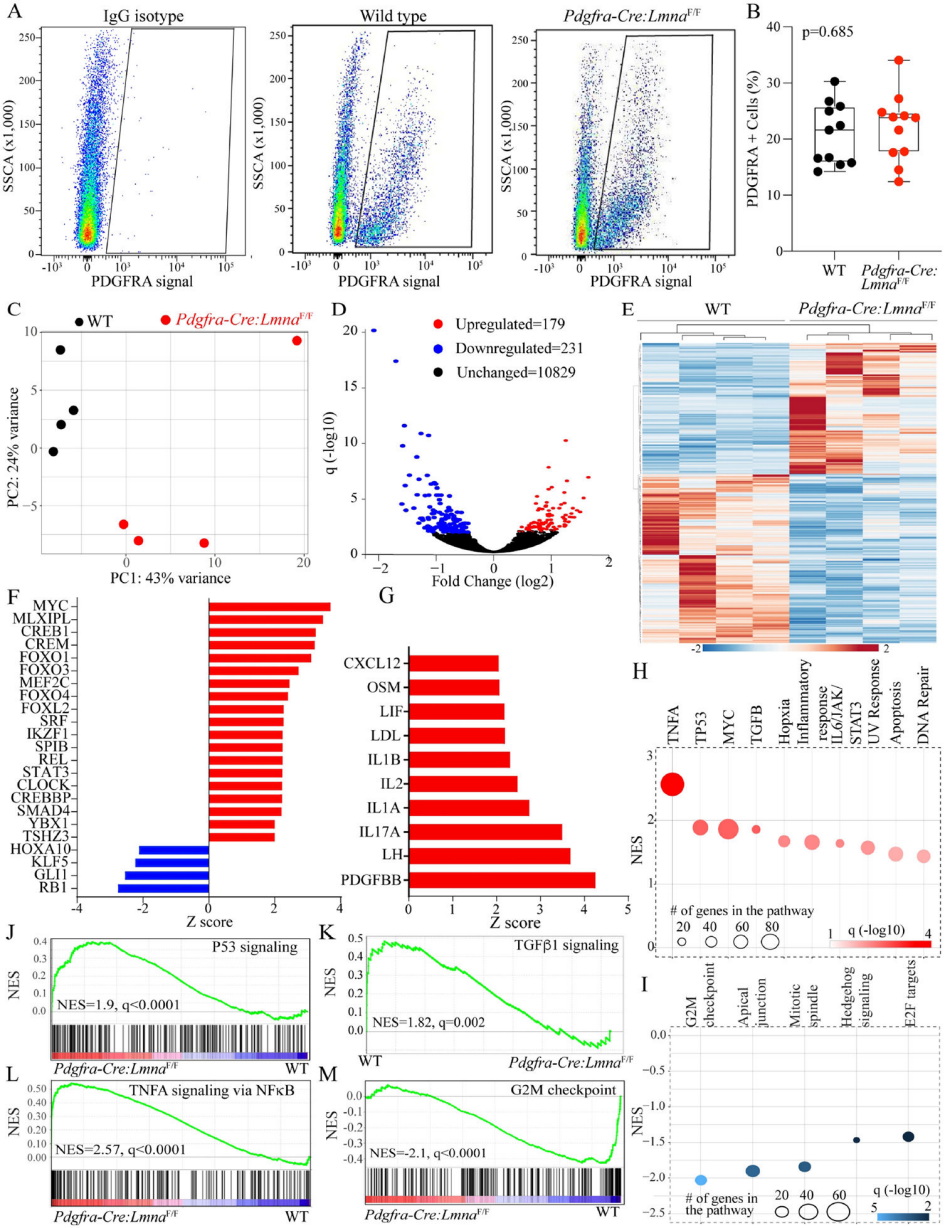
Differentially expressed genes, dysregulated transcriptional regulators, and biological pathways in the LMNA-deficient cardiac fibroblasts. (A) Fluorescence-activated cell sorting (FACS) panels showing the gating for IgG isotype and isolation of the cells expressing PDGFRA in the WT and *Pdgfra-Cre:Lmna*^F/F^ mice. (B) Quantitative data showing the percentage of the non-myocyte cells isolated based on the signaling from an antibody against PDGFRA in the 6-week-old WT and *Pdgfra-Cre:Lmna*^F/F^ mice. (C) Principle component analysis (PCA) of the cardiac fibroblast transcripts shows distinct separation between the WT and *Pdgfra-Cre:Lmna*^F/F^ genotypes. (D) Volcano plot showing the down-regulated (blue), upregulated (red), and unchanged (black) genes in the *Pdgfra-Cre:Lmna*^F/F^ as compared to the WT fibroblasts. (E) Heat map of the differentially expressed genes (DEGs) between the WT and *Pdgfra-Cre:Lmna*^F/F^ fibroblasts. (F) The bar chart represents the predicted transcriptional regulators of the DEGs in *Pdgfra-Cre:Lmna*^F/F^ fibroblasts. Red indicated predicted activation and blue predicted suppression. (G) The bar chart represents predicting growth and mitotic factors based on the DEGs in the *Pdgfra-Cre:Lmna*^F/F^ fibroblasts. (H) Biological pathways predicted to be activated in the *Pdgfra-Cre:Lmna*^F/F^ fibroblasts. (I) Biological pathways predicted to be suppressed in the *Pdgfra-Cre:Lmna*^F/F^ fibroblasts. (J-M) Plots representing gene set enrichment analysis (GSEA) predicting activation of the TP53, TNFA/NFκB, and TGFβ1 and suppression of the cell cycle G2M pathways.

Cardiac fibroblasts expressing PDGFRA but not the hematopoietic lineage markers were isolated by FACS from the WT and *Pdgfra-Cre:Lmna*^F/F^ mice and used for RNA extraction and sequencing. Cardiac fibroblasts from the *Pdgfra-Cre:Lmna*^W/F^ were not included as the phenotype in the latter group is largely similar to that in the *Pdgfra-Cre:Lmna*^F/F^ mice, except that it evolves later in life. To reduce the confounding effects of overt cardiac dysfunction on gene expression, the RNA-Seq experiments were performed in the RNA extracted from cardiac fibroblasts isolated from the 3- to 4-week-old mice with preserved LVFS (LVEF: 55.5 ± 4.8 vs. 50.8% ± 7.4% in the WT and *Pdgfra-Cre:Lmna*^F/F^ mice, respectively, *P* = 0.08). Approximately 20% of the lineage-negative sorted cells expressed PDGFRA in each genotype (WT: 20.9% ± 5.8% *vs. Pdgfra-Cre:Lmna*^F/F^ 22.0 ± 6.1, *P* = 0.685), which were isolated and used in the RNA-sequencing [Figure 8A and B].

Principle Component Analysis (PCA) showed distinct genotype-dependent separation of the samples [Figure 8C]. Analysis of the transcript showed differential expression of 410 genes between the WT and *Pdgfra-Cre:Lmna*^F/F^ mouse cardiac fibroblasts, which were comprised of 231 down-regulated and 179 upregulated genes [Figure 8D]. A heat map of the DEGs showed genotype-dependent expression of the dysregulated genes [Figure 8E]. Notable among the upregulated genes were *Egr2* (4.2-fold), *Ch25h* (3.7-fold), and *Nr4a2* (3.5-fold), encoding early growth response-2, cholesterol 25-hydroxylase, and nuclear receptor subfamily 4 group A member 2, respectively. The *Wfdc1*, *Col4a4*, and *Cxcl14*, encoding WAP four-sulfide core domain 1, collagen type IV, alpha 4 chain, and C-X-C motif chemokine ligand 14, respectively, comprised the list of topmost downregulated genes (~4 to 6-fold) in the *Pdgfra-Cre:Lmna*^F/F^ cardiac fibroblasts. Analysis of the DEGs predicted dysregulation of about two-dozen transcriptional regulators of gene expression, notable for the predicted activation of MYC, MLX1PL, CREB1, CREM, and FOXO transcription factors and predicted suppression of RB1, GLI1, KLFs, and HOXA10 in the *Pdgfra-Cre:Lmna*^F/F^ cardiac fibroblasts [Figure 8F]. Among the trophic and mitotic regulators of gene expression, the DEGs predicted activation of PDGFBB, LH, and several interleukins in the *Pdgfra-Cre:Lmna*^F/F^ cardiac fibroblasts [Figure 8G]. As for the biological pathways, the DEGs predicted activation of inflammatory and senescence pathways, including the TNFA, TP53, MYC, and TGFβ1, and suppression of the cell cycle pathways, such as the G2M checkpoint, mitotic spindle, and the E2F pathways [Figure 8H and I]. Moreover, GSEA predicted enrichment of the TP53, TNFA signaling via NFκB, and TGF β1 pathways and inhibition of the G2M checkpoint [Figure 8J-M].

### Increased expression levels of double-stranded DNA breaks (DSBs) and activation of the DDR Pathway:

**Figure 9.**
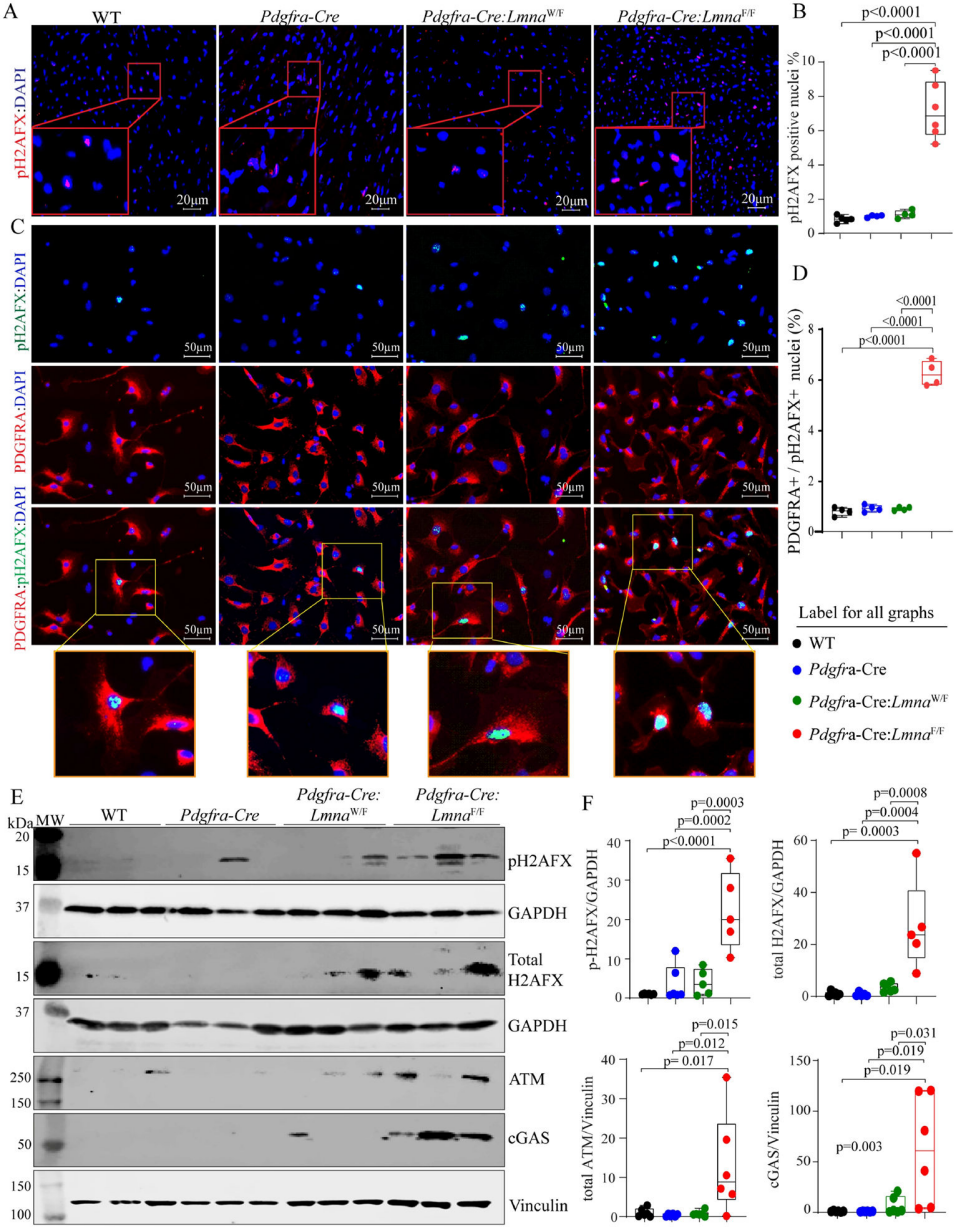
Activation of the DNA Damage Response (DDR) pathway. (A) Immunofluorescence panels showing expression of phospho-H2AFX, a marker for the double-stranded DNA breaks (DSBs) in the myocardial sections. (B) Quantitative data showing the percentage of nuclei staining positive for the expression of pH2AFX. (C) Immunofluorescence panels showing expression of phospho-H2AFX in isolated cardiac fibroblasts. Panels representing the expression of pH2AFX (green color), PDGFRA (red), and the overlay are presented along with enlarged inserts showing the expression of pH2AFX in the nuclei of isolated cardiac fibroblasts. (D) Graph depicting the percentage of the cells co-expressing PDGFRA and pH2AFX in the experimental and control groups. (E) Immunoblots showing expression of selected proteins involved in the DDR pathways, namely pH2AFX, total H2AFX, ATN, and CGAS, along with blot representing controls for the loading conditions, are shown in the WT, *Pdgfra-Cre*, *Pdgfra-Cre:Lmna*^W/F^, and *Pdgfra-Cre:Lmna*^W/F^ mice. (F) Quantitative data corresponding to the blots shown in panel C.

Given the predicted activation of the DDR pathway by the RNA-Seq data, expression, and levels of phospho-(p)H2AFX, a marker for the double-stranded DNA breaks (DSBs), were determined by immunofluorescence staining of the thin myocardial section, which showed an increased number of cell staining positive for pH2AFX in the *Pdgfra-Cre:Lmna*^F/F^ hearts [Figure 9A and B]. To determine whether pH2AFX expression was induced in cardiac fibroblasts, isolated non-myocyte cells were co-stained for pH2AFX and PDGFRA, and the number of cells expressing both markers was determined, which showed a significant increase in cells isolated from the *Pdgfra-Cre:Lmna*^F/F^ mouse hearts [Figure 9C and D]. To further corroborate the findings, expression levels of the pH2AFX and its upstream kinase ATM were determined by immunoblotting, which showed increased levels in the *Pdgfra-Cre:Lmna*^F/F^ hearts [Figure 9E and F]. Finally, because DSBs are sensed by cytosolic DNA sensor CGAS protein, its expression level was assessed by immunoblotting, which also showed increased levels in the *Pdgfra-Cre:Lmna*^F/F^ group [Figure 9E and F].

### Increased expression of the senescence markers:

**Figure 10.**
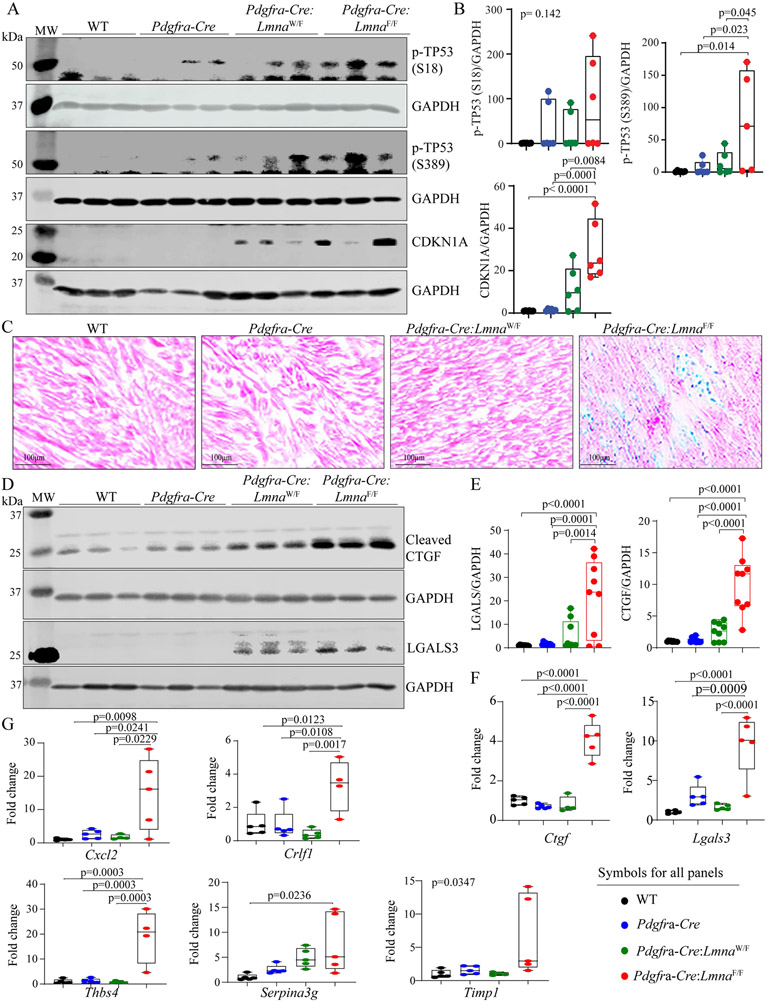
Expression of senescence markers. (A) Immunoblots showing levels of phospho-TP53 S18 and S389 proteins as well as the bonafide downstream target of activation of TP53, namely CDKN1A, in the experimental groups. (B) Quantitative data representing the blots shown in panel A. (C) Thin myocardial sections were stained for the expression of senescence-associated β-galactosidase, detecting its expression in the heart of 6-week-old *Pdgfra-Cre:Lmna*^F/F^ mice. (D) Immunoblots showing increased expression levels of CTGF (CCN2) and LGALS3, SASP markers, in the *Pdgfra-Cre:Lmna*^F/F^ mouse hearts. Quantitative data are shown in panels (E and F). (G) Transcript levels of selected senescence-associated secretory phenotype (SASP), quantified by RT-PCR, showing increased transcript levels in the *Pdgfra-Cre:Lmna*^F/F^ mouse hearts, as compared to other genotypes.

Given that the DDR pathway activates the cell senescence program by activating the TP53 and its bona fide target cell cycle inhibitor CDKN1A, expression levels of phosphoTP53 (residues Serine 18 and Serine 389) and CDKN1A were assessed by immunoblotting. Expression levels of pTP53 (Serine 18 and Serine 389) were increased in *Pdgfra-Cre:Lmna*^F/F^ mouse hearts at six weeks of age, as were CDKN1A protein levels [Figure 10A and B]. In conjunction with the increased expression levels of cell cycle inhibitors, expression of the senescence-associated β-galactosidase was induced in *Pdgfra-Cre:Lmna*^F/F^ mouse hearts [Figure 10C]. Likewise, expression levels of selected markers of senescence-associated secretory phenotype (SASP), namely CTGF (CCN2), and LGALS3, and as shown earlier TGFβ1, were increased in the hearts of six-week-old *Pdgfra-Cre:Lmna*^F/F^ mice, whereas their expression was either lower or not detected in the WT, *Pdgfra-Cre*, and six-week-old heterozygous (*Pdgfra-Cre:Lmna*^W/F^) mice [Figure 10D and E and Figure 5C and D]. In agreement with the protein levels, transcript levels of *Ctgf* (*Ccn2*) and *Lgals3* were also increased, indicating transcriptional activation of these genes [Figure 10F]. Finally, transcript levels of several additional genes involved in cell senescence were analyzed by RT-PCR. The findings were notable for increased transcript levels of proinflammatory genes *Crlf1*, *Cxcl2*, *Thbs4*, *Serpina3g*, and *Timp1* in the *Pdgfra-Cre:Lmna*^F/F^ mouse hearts but not in the WT or *Pdgfra-Cre* mouse hearts [Figure 10G].

## DISCUSSION

The findings highlight the contribution of cardiac fibroblasts to the pathogenesis of LMNA-associated DCM, as the deletion of the *Lmna* gene in fibroblasts leads to cardiac dilatation and dysfunction, cardiac arrhythmias, myocardial fibrosis, and apoptosis, a phenotype that resembles that of the DCM. The observed phenotype is not indistinct from DCM caused upon deletion of the *Lmna* gene in cardiac myocytes, albeit it is milder and occurs later in life^[19,23]^. Notably, systemic or myocyte-specific deletion of the *Lmna* gene leads to near-total mortality at three to four weeks of age, whereas the *Pdgfra-Cre:Lmna*^F/F^ mice survive to eight weeks^[19,23]^. Similarly, cardiac dysfunction is milder and occurs later in the *Pdgfra-Cre:Lmna*^F/F^ than *Myh6-Cre:Lmna* mice^[19,23]^. The findings are remarkable, as genetic cardiomyopathies are considered primarily a disease of cardiac myocytes, and the involvement of other cell types in the pathogenesis of cardiomyopathies is not well understood and generally considered a secondary event.

The expression of a DCM-like phenotype upon deletion of the *Lmna* gene in fibroblasts is in accord with the ubiquitous expression of the LMNA protein in various cell types, including cardiac fibroblasts^[8]^. Likewise, the observed phenotypic effects on cardiac function and arrhythmias likely reflect the cellular crosstalk between fibroblasts and myocytes, as well as the paracrine effects, including expression of the SASP. The latter mechanism is also in agreement with the paracrine effects resulting from the deletion of the placental growth factor in cardiac fibroblasts and the endothelial cells^[29]^. The findings also implicate increased DSBs, as evidenced by increased expression of the markers of the DSBs, activation of the DDR pathway, and increased expression of SASP, as the major mechanism in the pathogenesis of the phenotype.

The findings, along with the data in cardiac myocyte-specific models of laminopathies, denote activation of the DDR pathway as a cell-autonomous pathogenic consequence of LMNA deficiency in the heart^[19,23]^. Collectively, the data underscore the role of the LMNA protein as a guardian of genomic integrity. Increased expression levels of the markers of DSBs and activation of the DDR pathway are also in accord with the findings upon deletion of another nuclear member protein, namely TMEM43. Together, the findings suggest a possible shared mechanism involving DNA damage in the pathogenesis of DCM in nuclear envelopathies^[17]^.

The study has several limitations. The *Lmna* gene was deleted using the *Pdgfra-Cre* BAC transgenic deleter mice^[15]^. Although PDGFRA protein is abundantly expressed in cardiac fibroblasts, it is not an exclusive marker of cardiac fibroblasts, as it is also expressed in multiple mesenchymal tissues. No gross abnormalities were detected in other organs; however, the studies were primarily focused on evaluating the cardiac phenotype. Therefore, the possible presence of concomitant phenotypes in other organs cannot be excluded. The comprehensive metabolic panel did not show evidence of kidney or liver abnormalities or electrolyte disturbances. However, plasma glucose levels were reduced, and levels of the liver enzymes ALT and AST were modestly increased in the *Pdgfra-Cre:Lmna*^F/F^ mice. The latter changes were likely reflective of the smaller body size observed in the *Pdgfra-Cre:Lmna*^F/F^ mice. Overall, we surmise that the observed phenotype was cardiac-specific and likely the consequence of the deletion of the *Lmna* gene in cardiac fibroblasts but not due to abnormalities in other organs.

The PDGFRA is not expressed in mature cardiac myocytes; however, the locus is transcriptionally active in a small subset of cardiac myocytes during cardiogenesis^[16]^. In agreement with the previous data, using the *Pdgfra-Cre* mice led to the deletion of the *Lmna* gene in ~25% of cardiac myocytes in the *Pdgfra-Cre:Lmna*^F/F^ mice, and correspondingly, the LMNA protein levels in cardiac myocytes were reduced by ~30%. Therefore, one might surmise that deletion of the *Lmna* gene in a small subset of cardiac myocytes also contributed to the expression of the DCM-like phenotype in the *Pdgfra-Cre:Lmna*^F/F^ mice. The possibility cannot be excluded unambiguously. However, the heterozygous deletion of the *Lmna* gene in cardiac myocytes in the *Myh6-Cre-Lmna*^W/F^ mice also leads to about 30% to 50% reduction in the LMNA levels in cardiac myocytes, does not induce an early phenotype, in contrast to the *Pdgfra-Cre:Lmna*^F/F^ mice. Likewise, the *Pdgfra-Cre:Lmna*^F/F^ mice do not survive past eight weeks, whereas the *Myh6-Cre:Lmna*^W/F^, despite having a similar level of reduction in the LMNA protein levels in cardiac myocytes, survive up to 15 months^[19]^. In addition, the *Pdgfra-Cre:Lmna*^W/F^ mice, which show unchanged LMNA levels in cardiac myocytes but reduced LMNA in cardiac fibroblasts, also expressed a late-onset DCM-like phenotype. Furthermore, it merits noting that the gene expression analysis by RNA-Seq was performed in a subset of FACS-isolated cardiac fibroblasts, which expressed PDGFRA but not the hematopoietic lineage markers. The findings of the RNA-Seq data also pointed to the activation of the TP53 and NFκB and suppression of the cell cycle pathways, which agree with the other molecular, biological, and histological findings in this model. In support of the RNA-Seq data, the expression of pH2AFX, a bona fide marker of double-stranded DNA breaks, was increased in the isolated cardiac fibroblasts. Collectively, the data support the notion that the deletion of the *Lmna* gene in cardiac fibroblasts was the main determinant of the ensuing phenotype in the *Pdgfra-Cre:Lmna*^F/F^ mice. Nevertheless, the contribution of partial LMNA deficiency in a fraction of the cardiac myocytes to the phenotypic expression of a DCM-like phenotype in the *Pdgfra-Cre:Lmna*^F/F^ cannot be excluded. New approaches and models would be necessary to specifically delete the *Lmna* gene in cardiac fibroblasts and exclude the confounding effects of the LMNA-deficiency in other cell types and organs.

In conclusion, deficiency of the LMNA protein in cardiac fibroblasts contributes to the pathogenesis of the LMNA-associated DCM by activating the DDR pathway in response to increased DSBs and inducing fibroblast-senescence. The findings indicate a multi-cellular basis for the phenotype in LMNA-associated DCM. The findings of the present study, in conjunction with the previous data in myocyte-specific models, identify the DSBs as a major mechanism in the pathogenesis of LMNA-associated DCM and denote the LMNA protein as a guardian of genomic stability.

## Supplementary Material

Supplementary material

## Figures and Tables

**Table 1.** Echocardiographic phenotype at six weeks of age

	WT	*Pdgfra-Cre*	*Pdgfra-Cre:Lmna* ^W/F^	*Pdgfra-Cre:Lmna* ^F/F^	*P* (ANOVA)
N	11	10	10	12	NA
M/F	6/5	5/5	5/5	6/6	0.995^^^
Age (days)	41.27 ± 1.60	42.3 ± 2.10	43.50 ± 4.20	39.01 ± 1.03	0.058
Body weight (g)	21.59 ± 3.40	22.58 ± 3.00	21.83 ± 2.80	7.64 ± 1.80^#^*^&^	< 0.0001
HR (bpm)	468 ± 28	477 ± 50	516 ± 68	468 ± 53	0.292
AWT (mm)	0.57 ± 0.07	0.64 ± 0.09	0.55 ± 0.04	0.43 ± 0.06^#^*^&^	< 0.0001
LVPWT (mm)	0. 58 ± 0.69	0. 61 ± 0.11	0.55 ± 0.05	0.41 ± 0.06^#^*^&^	< 0.0001
LVEDD (mm)	3.68 ± 0.17	3.62 ± 0.30	3.49 ± 0.36	2.97 ± 0.28^#^*^&^	< 0.0001
LVEDDI (mm/g)	0.17 ± 0.02	0.16 ± 0.02	0.16 ± 0.01	0.40 ± 0.08^#^*^&^	< 0.0001
LVESD (mm)	2.20 ± 0.16	2.16 ± 0.28	2.21 ± 0.35	2.12 ± 0.35	0.915
LVESDI (mm/mg)	0.10 ± 0.01	0.10 ± 0.01	0.10 ± 0.01	0.29 ± 0.07^#^*^&^	< 0.0001
FS (%)	40.20 ± 3.49	40.39 ± 3.71	37.07 ± 4.27	28.82 ± 6.59^#^*^&^	< 0.0001
LV Mass (mg)	59.28 ± 13.09	55.16 ± 10.89	47.42 ± 11.65	25.14 ± 5.46^#^*^&^	< 0.0001
LVMI (mg/g)	2.54 ± 0.32	2.64 ± 0.63	2.17 ± 0.46	3.34 ± 0.48^#^*^&^	< 0.0001

M/F: Male/Female; g: gram; mg: milligram; mm: millimeter; HR: heart rate; bpm: beats per minute; AWT: anterior wall thickness; LVPWT: left ventricular posterior wall thickness; LVEDD: left ventricular end-diastolic diameter; LVEDDi: LVEDD indexed to body weight; FS: fractional shortening; LVM: left ventricular mass; LVMI: LVM indexed to the body weight.

^: denotes *X*^2^: test

#: denotes *P* < 0.05 *vs.* WT

* denotes *P* < 0.05 vs. *Pdgfra-Cre*

&: denotes *P* < 0.05 *vs. Pdgfro-Cre:Lmna*^W/F^. *P*-values were obtained by one-way ANOVA followed by Bonferroni pairwise comparisons.

**Table 2.** Echocardiographic indices of cardiac size and function in 15-month-old *Pdgfra-Cre:Lmna*^W/F^ mice

	WT	*Pdgfra-Cre*	*Pdgfra-Cre:Lmna* ^W/F^	*P* (ANOVA)
N	11	11	14	
M/F	4/7	6/4	7/7	0.670^^^
Age (month)	14.33 ± 2.01	14.50 ± 0.81	15.56 ± 2.16	0.125
Body weight (g)	40.16 ± 4.97	39.151 ± 5.52	35.77 ± 4.21	0.329
HR (bpm)	468 ± 45	459 ± 37	421 ± 25	0.582
AWT(mm)	0.68 ± 0.08	0.63 ± 0.03	0.67 ± 0.10	0.356
LVPWT(mm)	0.75 ± 0.08	0. 64 ± 0.07	0.70 ± 0.07	0.080
LVEDD (mm)	4.08 ± 0.38	4.15 ± 0.44	4.25 ± 0.38	0.534
LVEDDI(mm/g)	0.10 ± 0.01	0.11 ± 0.01	0.12 ± 0.01^#^*	0.0003
LVESD (mm)	2.46 ± 0.40	2.58 ± 0.37	2.89 ± 0.41^#^*	0.036
LVESDI (mm)	0.07 ± 0.01	0.06 ± 0.01	0.08 ± 0.01^#^*	< 0.0001
FS (%)	39.30 ± 5.67	38.00 ± 3.90	32.20 ± 4.91^#^*	0.002
LV Mass (mg)	84.00 ± 13.56	77.02 ± 19.60	88.70 ± 26.01	0.460
LVMI (mg/g)	2.09 ± 0.23	1.94 ± 0.40	2.46 ± 0.50*	0.020

M/F: Male/Female; g: gram; mg: milligram; mm: millimeter; HR: heart rate; bpm: beats per minute; AWT: anterior wall thickness; LVPWT: left ventricular posterior wall thickness; LVEDD: left ventricular end-diastolic diameter; LVEDDi: LVEDD indexed to body weight; FS: fractional shortening; LVM: left ventricular mass; LVMI: LVM indexed to the body weight.

^: denotes *X*^2^: test

#: denotes *P* < 0.05 *vs.* WT

* denotes *P* < 0.05 *vs. Pdgfra-Cre. P*-values were obtained by one-way ANOVA followed by Bonferroni pairwise comparisons.
