# Supplementary material for: Deletion of the Lmna gene in fibroblasts causes senescence-associated dilated cardiomyopathy by activating the double-stranded DNA damage response and induction of senescence-associated secretory phenotype
Source: J Cardiovasc Aging. Author manuscript; Available in PMC 2022 Jul 25. (PMC9311325; doi:10.20517/jca.2022.14)
Supplement: Supplementary material [file NIHMS1816151-supplement-Supplementary_material.pdf]

Supplementary Material

Deletion of the *Lmna* Gene in Fibroblasts Causes Senescence-Associated Dilated Cardiomyopathy by Activating the Double-Stranded DNA Damage Response and Induction of Senescence-Associated Secretory Phenotype

Leila Rouhi et al.

Online Table 1

List of oligonucleotide primers

A. Genotyping primers

Gene	Forward sequence	Reverse sequence
Lmna Flox	TCGAGGCTCTTCTCAACTCC	CTCTCCTCTGAAGTGCTTGGA
Cre recombinase	GCGGTCTGGCAGTAAAAACTATG	GTGAAACAGCATTGCTGTCACTT
Internal control	CTAGGCCACAGAATTGAAAGATCT	GTAGGTGGAAATTCTAGCATCATCC

B. Antibodies

Antibodies	Concentration	Supplier	Catalog number
Anti-mouse IgG HRP linked antibody	1:4000 (IB)	Cell Signaling Technology	7076
Anti-rabbit IgG HRP linked antibody	1:2000 (IB)	Cell Signaling Technology	7074
Goat anti-Mouse IgG, Alexa Fluor 488	1:1000 (IF)	Invitrogen	A11029
Goat anti-Rabbit IgG, Alexa Fluor 594	1:1000 (IF)	Invitrogen	A11005
GAPDH	1:1000 (IB)	Cell Signaling Technology	8245
LMNA/C (E-1)	1:50 (IF)	Santa Cruz	376248
LMNA	1:1000 (IB)	Abcam	26300
PDGFRα	1:200 (IF)	Cell Signaling Technology	3174S
TP53	1:250 (IB)	Santa Cruz	6243
p-P53(S15)	1:1000 (IB)	Cell Signaling Technology	9284
p-P53(S392)	1:1000 (IB)	Cell Signaling	9281

		Technology	
CDKN1A	1:500 (IB)	Pharmingen	556431
PLIN1	1:200 (IF)	Cell Signaling Technology	9349
cGAS (D3080)	1:1000 (IB)	Cell Signaling Technology	31659
GALECTIN3 (LGALS3)	1:1000 (IB)	Abcam	53082
ATM	1:1000 (IB)	Cell Signaling Technology	2873
pH2AFX	1:1000 (IB), 1:200 (IF)	EMD Millipore	05-636
H2AX	1:1000 (IB)	Millipore Sigma	AB10022
CTGF		Santa Cruz	365970
PCM1	1:250	Sigma	HPA023370

C. Oligonucleotide primers used in qPCR reactions

Name	Sequence
Gapdh	Forward: AACTTTGGCATTGTGGAAGG<br>Reverse: GGATGCAGGGATGATGTTCT
Tgfb1	Forward:TGGAGCAACATGTGGAACTC<br>Reverse: GTCAGCAGCCGGTTACCA
Tgfb2	Forward: AGGAGTGGCTTCACCACAAAGACA<br>Reverse:ATTAGACGGCACGAAGGTACAGCA
Timp1	Forward: CATGGAAAGCCTCTGTGGATA<br>Reverse: CTCAGAGTACGCCAGGGAAC
Dgat1	Forward: GAGGTGCGAGACGCGG<br>Reverse: ATGGCACCTCAGATCCCAGTA
Bnip3	Forward: TCCTGGGTAGAACTGCACTTC<br>Reverse: GCTGGGCATCCAACAGTATTT
Gadd45g	Forward: GGGAAAGCACTGCACGAACT<br>Reverse: AGCACGCAAAAGGTCACATTG
Gadd45b	Forward: CAACGCGGTTCAGAAGATGC<br>Reverse: GGTCCACATTCATCAGTTTGGC
Bcl2l11	Forward: CCCGGAGATACGGATTGCAC<br>Reverse: GCCTCGCGGTAATCATTTGC
Bcl2	Forward: CACCCCTGGTGGACAACATC<br>Reverse:GTTCCACAAAGGCATCCCAGC
Cxcl2	Forward: TCCAGAGCTTGAGTGTGACG<br>Reverse: GCAAACTTTTTGACCGCCCT
Crlf1	Forward: AGCCCACACAGCTGTAATCA<br>Reverse: GGTCCAGTAGAGCCCCTCA
Thbs4	Forward: GACCCTGCCCAGATGGTTAC<br>Reverse: TCACATCTGAAACCCGGAGC
Serpina3g	Forward: AATTCAGCATGCAGTGGAGAGA<br>Reverse:GGGAGACACCAGCCATTATTCT
Pcolce	Forward: GGGCACTGAGCACCAGTTTT

	Reverse: GCTGATGCCTGGTGGGTAAT
Col5a2	Forward: GTACCACGGGCAAAGAGGAA<br>Reverse: CTTTTCCTGGTGTACCCGCT
Postn	Forward: AGAGAAATCCCTCACGACA<br>Reverse: GTTGGTGCAAACAAGGTCCA
Lgals3	Forward: TAATCAGGTGAGCGGCACAG<br>Reverse: GCTAAGGCATCGTTAAGCGAAA
Bax	Forward: ACAGGGGCCTTTTTGCTACA<br>Reverse: CACTCGCTCAGCTTCTTGGT
Bbc3(Puma)	Forward: GAGACAAGAAGAGCAGCATCG<br>Reverse: TAGTTGGGCTCCATTTCTGG

D. TaqMan probes

Gene	TaqMan Assay ID
Gapdh	Mm99999915_g1
Col1a1	Mm00801666_g1
Col3a1	Mm00802300_m1
Adipoq	Mm00456425_m1
Apoe	Mm01307193_g1
Pparg	Mm00440940_m1
Cebpa	Mm00514283_s1
Ctgf	Mm01192932-g1
Dgat2	Mm00499536_m1

Online Table 2

Comprehensive Metabolic Panel

Assay	Units	Wild Type	*Pdgfra-Cre:Lmna*$^{W/F}$	*Pdgfra-Cre:Lmna*$^{F/F}$	p
**N**		5	5	5	
**CHOL**	**Mg/dL**	93.5±13.5	72±10	70.3±6.5	0.06453
**TRIG**	**Mg/dL**	214±47.6	139.7±0.5	101.5±0.5	0.10896
**ALT**	**U/L**	32.4±5.7	20.4±4.1	61±27	0.06318
**AST**	**U/L**	59.6±20.8	61.2±30.2	151.6±95.9	0.07704
**ALP**	**U/L**	217.6±27.2	223.2±58.7	231.8±48.9	0.61224
**GLU**	**Mg/dL**	212.8±10.8	221.2±22.2	135.6±35	0.00338
**PHOS**	**Mg/dL**	9.7±0.6	11.15±0.2	6.2±4.3	0.28307
**Ca**	**Mg/dL**	10.9±0.1	10.8±0.4	10.8±0.2	0.32224
**TP**	**g/dL**	4.7±0.1	4.5±0.05	4.5±0.2	0.15975
**ALB**	**g/dL**	2.9±0.2	2.8±0.1	2.9±0.1	0.74912
**GLOB**	**g/dL**	1.7±0.1	1.6±0	1.6±0.05	0.40097
**A/G**	**—**	1.8±0.2	1.8±0	1.9±0	0.64559
**BUN**	**Mg/dL**	27±0.7	23±3.7	31.6±7.3	0.24850
**CREAT**	**Mg/dL**	0.2±0	0.2±0	0.1±0	—
**B/C**	**—**	135±3.5	115±1.5	—	—
**TBIL**	**Mg/dL**	0.16±0.03	0.14±0.02	0.24±0.08	0.08348
**Na**	**mEq/L**	150±0.8	150.4±1.5	153.8±0.9	0.04478
**K**	**mEq/L**	8.08±0.2	9.0±0.9	8.2±0.4	0.53160

**Na/K**	—	18.574±0.4	16.7±1.6	18.7±1.1	0.78760

Abbreviations: *Pdgfra-Cre:Lmna*$^{W/F}$: Mice with heterozygous deletion of the *Lmna* gene; *Pdgfra-Cre:Lmna*$^{F/F}$: mice with homozygous deletion of the *Lmna* gene; CHOL: cholesterol; TRIG: Triglyceride, ALT: Alanine aminotransferase; AST: Aspartate aminotransferase; ALP: Alkaline phosphatase; GLU: Glucose; Phos: Phosphorous; Ca: Calcium; TP: Total protein; ALB: Albumin; A/G: Albumin/Globulin ratio; GLOB: Globulin; BUN: Blood urea nitrogen; CREAT: Creatinine; TBIL: total bilirubin; Na: Sodium; K: Potassium; Na/K: Sodium/Potassium ratio.
